# The effect of internal salary incentives based on insurance payment on physicians’ behavior: experimental evidence

**DOI:** 10.1186/s12913-023-10408-8

**Published:** 2023-12-14

**Authors:** Xing Li, Jiali Teng, Xinyan Li, Xing Lin, Youli Han

**Affiliations:** School of Public Health, Capital Medical University, No.10 Xitoutiao, Youanmenwai Street, Fengtai District, Beijing, 100069 China

**Keywords:** Salary level, Salary composition, Physicians’ behavior, Experimental economics

## Abstract

**Background:**

Understanding how physicians respond to payment methods is crucial for designing effective incentives and enhancing the insurance system. Previous theoretical research has explored the effects of payment methods on physician behavior based on a two-level incentive path; however, empirical evidence to validate these theoretical frameworks is lacking. To address this research gap, we conducted a laboratory experiment to investigate physicians’ behavioral responses to three types of internal salary incentives based on diagnosis-related-group (DRG) and fee-for-service (FFS).

**Methods:**

A total of 150 medical students from Capital Medical University were recruited as participants. These subjects played the role of physicians in choosing the quantity of medical services for nine types of patients under three types of salary incentives—fixed wage, constant fixed wage with variable performance wage, and variable fixed wage with variable performance wage, of which performance wage referred to the payment method balance under FFS or DRG. We collected data on the quantities of medical services provided by the participants and analyzed the results using the Friedman test and the fixed effects model.

**Results:**

The results showed that a fixed wage level did not have a significant impact on physicians’ behavior. However, the patients benefited more under the fixed wage compared to other salary incentives. In the case of a floating wage system, which consisted of a constant fixed wage and a variable performance wage from the payment method balance, an increase in performance wage led to a decrease in physicians’ service provision under DRG but an increase under FFS. Consequently, this resulted in a decrease in patient benefit. When the salary level remained constant, but the composition of the salary varied, physicians’ behavior changed slightly under FFS but not significantly under DRG. Additionally, patient benefits decreased as the ratio of performance wages increased under FFS.

**Conclusions:**

While using payment method balance as physicians’ salary may be effective in transferring incentives of payment methods to physicians through internal compensation frameworks, it should be used with caution, particularly when the measurement standard of care is imperfect.

**Supplementary Information:**

The online version contains supplementary material available at 10.1186/s12913-023-10408-8.

## Introduction

Understanding how physicians respond to financial incentives is crucial for designing effective healthcare markets [1–3]. Further investigations have often focused on physicians’ behavioral reactions to various payment methods [4, 5]. In terms of the effects of payment methods on physicians’ behavior, the types of incentive paths are two: direct and indirect. The direct incentive path involves physicians receiving direct compensation from medical insurance. However, when physicians are employed by medical organizations and receive salaries, as is the case for a significant proportion of physicians in the United States [6], the relationship between payment methods and physicians’ behavior becomes multi-level (i.e., indirect incentive path). Previous empirical [7–10] and experimental studies [11–17] primarily have focused on investigating physicians’ responses to different forms of remuneration, such as fee-for-service (FFS), capitation (CAP), salary, and pay-for-performance (P4P), based on a direct incentive path. However, research on the indirect incentive path, specifically examining the relationship between insurance payments and physicians’ behavior when they receive salaries from medical organizations, is lacking.

Previous theoretical research has discussed the effects of payment methods on physicians’ behavior based on a two-level incentive path. Conrad et al. [18, 19] explored the multi-level incentive structure of payment methods, which includes the incentives of service purchasers to medical organizations and those of medical organizations to individual physicians. Building upon this, Conrad [20] developed a theoretical framework that considered payment incentives as a two-tier system: the first tier involved payments made by private or public medical insurance to service provider organizations (external incentives), and the second tier involved compensation provided to physicians by the provider organizations (internal incentives). Robinson [21] argues that the discrepancy in incentive approaches between medical insurance payments to physicians’ organizations and payments made by physicians’ organizations to individual physicians leads to an incentive mismatch, thereby hindering the effective transmission of the incentive effects of the medical insurance payment methods to physicians. When medical insurance payments to provider organizations are aligned with the salary incentives of individual physicians within those organizations, physicians are more likely to receive appropriate financial compensation and deliver effective medical care to patients [22]. While some studies have examined changes in physicians’ behavior when they are employed by hospitals [6, 23, 24], a gap in the literature remains regarding the relationships between payment methods and internal compensation within hospitals and how these factors influence physicians’ behavior.

In China, most physicians are employed by hospitals. The effect of payment methods on physicians’ behavior is a two-tier incentive system. The first tier refers to payments from the national basic medical insurance fund to hospitals, and the main medical insurance payment methods include FFS and diagnosis-related-group (DRG). The second tier refers to the internal physician compensation incentives within hospitals. Specifically, the salary system for physicians in Chinese hospitals has been the post-performance payment system (PPPS) since 2006, which consists of four main components [25]: position wage, reflecting the responsibilities and requirements of the position; seniority wage, reflecting the qualifications and seniority of jobs; performance wage, reflecting job performance and contributions; and allowance and subsidies, reflecting compensation for special circumstances. Overall, these four components can be divided into two parts: fixed salary (reflecting qualifications, responsibilities, and job requirements) and performance salary (reflecting individual contributions and performance). The performance salary is the primary source of income for physicians; hospitals compensate physicians based on performance evaluation outcomes.

An individual’s salary typically comprises two components: a fixed wage and a variable wage. Based on simple standard agency models, a fixed wage ensures the agent’s participation, whereas the variable part induces incentives [26, 27]. Experimental studies on fairness perception [28] and gift-exchange [29] have provided evidence that higher fixed wages lead to increased effort levels. Manthei and Mohnen [30] demonstrated that fixed wages had a notable effect on individual behavior, and linear incentive schemes were effective when the fixed wage was either relatively low or very high. Kirstein [31] conducted a study to assess the effectiveness of fixed wages and performance incentives (i.e., rewards and punishments). The findings indicated that in terms of performance, neither rewards nor punishments were significantly more effective than fixed wages alone. Moreover, some studies have investigated the effect of P4P, which builds upon payment methods such as FFS, CAP, DRG, and salary, on physicians’ behavior and quality of care [16, 17, 32, 33]. These studies demonstrate the trend of comprehensively considering payment methods and internal salary incentives to explore the effects of payment methods on physicians’ behavior. In summary, while several studies have explored the effects of payment methods or salary incentives on individual or physician behavior, few have analyzed the effects of payment methods on physicians’ behavior when considering internal salary incentives within medical organizations, namely fixed wages and performance wages.

The FFS has long been a primary medical insurance payment method worldwide. To address escalating healthcare expenditure, the Chinese government has been actively implementing payment method reform in recent years, and the DRG payment has emerged as a significant alternative to the traditional FFS payment. As the payment method reform progressed, the performance salary assessment and distribution system in hospitals gradually shifted from an economic profit performance assessment model to a medical insurance payment performance assessment model. The economic profit performance assessment model refers to the calculation of physicians’ salary based on a certain proportion of a hospital’s income minus expenses. Under the medical insurance payment performance assessment model, the utilization of medical insurance payments, that is, “the payment method balance (medical insurance payment–disease medical costs)”, is incorporated into the assessment of physicians’ performance and can influence their salary [34]. Some hospitals have gradually adopted this model [35] and distributed a portion of the surplus medical insurance payment as part of the physicians’ performance salaries [36]. Moreover, in 2021, the Chinese government issued a series of policy documents [37, 38] proposing to explore medical expense control, improve the efficiency of medical insurance fund utilization through payment method reform, and provide performance incentives to physicians by using surplus medical insurance payment. This prompts a critical inquiry into the correlation between payment methods, potential alterations in physicians’ salary levels and compositions, and the subsequent impact on physicians’ behavior.

Empirical research on the effects of payment methods on physicians’ behavior faces certain difficulties in data acquisition, comparative analysis of results, and generalization due to variations in healthcare systems, the complexity of medical service provision, and the heterogeneity of physicians’ intrinsic motivations. Economic experiments provide methodological support for addressing these issues [11]. In recent years, an increasing number of studies have used laboratory experiments to investigate physicians’ responses to payment methods [11–17]. To gain insights into the policy effects of the ongoing payment method reform in China, we conducted a controlled laboratory experiment to examine physicians’ behavioral responses to different salary incentives using two payment methods: FFS and DRG. Taking reference to and drawing lessons from the studies of Brosig-Koch et al. [15–17], we modified the experimental design and extended their research. In our previous experiment, we examined the effects of payment methods, including DRG, FFS, and mixed payment schemes, on physicians’ behavior within the framework of the direct incentive path for payment methods [39]. Based on our previous study, we designed this experiment to investigate how physicians react to different types of compensation under the indirect incentive path for payment methods, which has rarely been explored. The compensation design for physicians included the fixed wage and a mixed payment scheme combining the fixed wage and payment method balance; the latter is called the floating wage system. This floating wage system can be classified into two scenarios: changes in the total salary and changes in salary composition. Our study aimed to address two primary questions: whether the incentive effect of payment methods could be effectively conveyed to physicians through a floating wage system based on payment method balance, and how physicians respond to different wage levels and compositions under the floating wage system.

## Methods

### Experimental setting and conditions

In the experiment, each subject i, in the role of a physician, was asked to choose the quantity of medical service, $q \in \left\{\mathrm{0,1},\dots 10\right\}$, for nine different types of patients under given conditions. The nine types of patients differed in three illnesses: $k \in \left[A, B, C\right]$and severity of these illnesses, j $\in [mild\left(1\right), moderate\left(m\right),severe(h)]$. All patients were assumed to be covered by medical insurance and accept any medical services provided by physicians. According to the quantity of medical services provided by physicians, hospitals received a certain payment R and incurred costs ${C}_{kj}\left(q\right) = 0.1\cdot {q}^{2}$ [40] for treating patients. Furthermore, the physicians’ quantity choices determined not only their own profit ${\pi }_{kj}^{i}$ but also the patient benefit ${B}_{kj}\left(q\right)$. Physicians’ profits and patient benefits were measured in monetary terms. The accumulated physicians’ profit served as remuneration for the subjects, while the patient’s benefit was donated to charitable institutions to assist real patients [11, 15–17].

The patient benefit was designated as ${B}_{kj}\left(q\right) = {B}_{kj}\left({q}^{*}\right) - \theta \left|q - {q}^{*}\right|$ [16]. q^*^ refers to the optimal quantity to obtain the maximum patient benefit; θ refers to the marginal patient benefit, ${\theta }_{A} = {\theta }_{B} =1$, ${\theta }_{C}=2$. When the severity of illness varies between mild, moderate, and severe, the optimal quantity q^*^ is 3, 5, and 7, respectively. Maximum patient benefit differed for different illnesses: ${B}_{Aj}\left({q}^{*}\right) = 10$, ${B}_{Bj}\left({q}^{*}\right) = 15$, and ${B}_{Cj}\left({q}^{*}\right) = 20$. Using the optimal quantity q^*^ as the benchmark, we could determine whether the quantity of medical services chosen by the subjects was undersupplied or oversupplied.

Physician salary (profit): ${\pi }_{kj}^{i} =t + \alpha \left[R - {C}_{kj} \left(q\right)\right]$ . The physicians’ salary consists of two parts: fixed wage *t* and performance wage $\alpha \left[R - {C}_{kj} \left(q\right)\right]$. We assumed that the physicians’ performance wage was mainly determined by the payment method balance, *R - C*. $\alpha \in \left[\mathrm{0,1}\right]$ represents the proportion of the payment method balance used for issuing performance wage. $\alpha =0$ indicates that the physicians’ salary was a pure fixed wage; $0< \alpha <1$ indicates that the physician salary included both fixed and performance wages; and $\alpha =1$ indicates that the entire payment method balance was used as a performance wage.

Table 1 lists the experimental conditions, including the three parts for each group. Our experiment included three variables: payment method, wage level, and wage composition (the respective proportions of fixed and performance wages). We adopted a between-subjects design for payment methods. The payment method was DRG (FFS) in groups II, and III (IV, and V). For wage level and composition, we adopted a within-subjects design for the five groups. Participants were required to choose the quantity of medical services for patients under different salary incentives.

**Table 1:** Experiment condition

Payment	Physicians’ salary			Group	q^
Fixed Wage	t	2t	3t	I	-
Floating Wage under DRG	DRG-Lev-25%	DRG-Lev-50%	DRG-Lev-75%	II	0
	DRG-Com-25%	DRG-Com-50%	DRG-Com-75%	III	0
Floating Wage under FFS	FFS-Lev-25%	FFS-Lev-50%	FFS-Lev-75%	IV	10
	FFS-Com-25%	FFS-Com-50%	FFS-Com-75%	V	10

Table 1 shows the experimental conditions. t, 2t, and 3t refer to three levels of fixed wage. DRG (FFS)-Lev-25%, DRG (FFS)-Lev-50%, DRG (FFS)-Lev-75% refer to different level of floating wage system that consists of constant fixed wage and variable performance wage in group II (IV). DRG (FFS)-Com-25%, DRG (FFS)-Com-50%, DRG (FFS)-Com-75% refer to the different compositions of floating wage system that consists of variable fixed wage and variable performance wage in group III (V). q^, the chosen quantity to maximize physicians’ profit, is 0 (10) under groups II, and III (IV, and V)

#### Fixed wage

Group I was designed to investigate how physicians responded to different levels of fixed wage. Given that the salary of Chinese physicians is relatively low compared to other countries, such as the United States and Canada, where the ratio of physicians’ salary to GDP per capita is higher, we deemed it necessary to increase the salary of Chinese physicians by 2–3 times [41]. Hence, we established three levels of fixed wage: one low level (t) and two high levels (2t and 3t). In our previous study [39], we found that the average profit for physicians under FFS and DRG payment was 7.98 and 8.28, respectively. Taking a rounded average of eight as the basic remuneration that physicians could receive, we considered this to be the fixed wage. Therefore, physicians’ fixed wages in the three parts of group I were set to 8, 16, and 24.

#### Floating wage system

We aimed to investigate the effect of the floating wage level (i.e., constant fixed wage t with variable performance wage) on physicians’ behavior in groups II, and IV. To ensure the comparability of patient benefit and physicians’ profit, the performance wages were set at 25%, 50%, and 75% of the payment method balance, respectively, in the three parts of group II (IV). The salary incentives in group II (IV) were labeled DRG-Lev-25%, DRG-Lev-50%, and DRG-Lev-75% (FFS-Lev-25%, FFS-Lev-50%, and FFS-Lev-75%), respectively.

In groups III and V, the salary composition varied, with the fixed wage being adjustable and performance wages set at 25%, 50%, and 75% of the payment method balance, respectively, in each part of the experiment. The salary incentives in groups III (V) were labeled DRG-Com-25%, DRG-Com-50%, and DRG-Com-75% (FFS-Com-25%, FFS-Com-50%, and FFS-Com-75%), respectively. In each part of group III (V), the physicians’ total salary remained constant; it was equal to the physicians’ profit in the third part, namely, DRG-Com-75% (FFS-Com-75%).

In groups II, and III, hospitals received a lump-sum payment per patient type based on k and j. The specific lump-sum payments (LS) for patient types A_l_, A_m_, A_h_, B_l_, B_m_, B_h_, C_l_, C_m_, and C_h_ were 5.73, 9.55, 13.37, 6, 10, 14, 6.3, 10.5, and 14.7, respectively [39]. Physicians’ profits were ${\pi }_{kj}^{i} =t + \alpha \left(LS -0.1\cdot {q}^{2}\right)$. *q*^^^, the chosen quantity to maximize physicians’ profits, was 0. According to our calculations, the physicians’ average maximum profits in the three parts of group II were 10.50, 13.01, and 15.51, respectively. Compared with DRG-Lev-25%, physicians’ maximum profits under DRG-Lev-50% and DRG-Lev-75% increased by 23.37% and 46.73%, respectively, on average. The average ratios of performance wages to physicians’ total salaries were 15.62%, 25.34%, and 31.76% for DRG-Lev-25%, DRG-Lev-50%, and DRG-Lev-75%, respectively. In group III, the average ratios of performance wages to physicians’ total salaries were 10.59%, 21.17%, and 31.76% under DRG-Com-25%, DRG-Com-50%, and DRG-Com-75%, respectively.

In groups IV and V, the hospital received a payment p for each service provided by the physicians, and the total payment was $R = {\text{pq}}$. We set *p* for illnesses A, B, and C to 1.91, 2, and 2.1, respectively [39]. Accordingly, physicians’ profits were ${\pi }_{kj}^{i} =t + \alpha \left(pq -0.1\cdot {q}^{2}\right)$, and *q*^^^ was 10. In group IV, the physicians’ average maximum profits in each part were 10.51, 13.02, and 12.53, respectively. Compared with FFS-Lev-25%, the physicians’ maximum profits under FFS-Lev-50% and FFS-Lev-75% increased by 23.84% and 47.69%, respectively, on average. The average ratios of performance wages to physicians’ total salaries were 16.26%, 27.16%, and 35.06% under FFS-Lev-25%, FFS-Lev-50%, and FFS-Lev-75%, respectively. In group V, the average ratios of performance wages to physicians’ total salaries were 11.69%, 23.37%, and 35.06% for FFS-Com-25%, FFS-Com-50%, and FFS-Com-75%, respectively.

### Experimental protocol

The computerized experiment was programmed using Z-tree [42] and conducted in June 2021. We used G*power 3.1.9.7 [43] and set the significance level to 0.05, power to 0.80, effect size to 0.25 [44], correlation to 0.50, and non-sphericity correction to 1.0 to calculate the sample size; the results showed that at least 28 physicians were required per group. Referring to the sample size of related economic experiments on payment systems [11, 15], we decided on a sample size of 30 subjects per group. We recruited 150 medical students from Capital Medical University and randomly divided them into five groups (Table 1). To prevent order effects, we randomly assigned 30 subjects in each group to six different sequences of experimental conditions. All subjects entered hospitals to fulfill clinical rotation requirements. These subjects had medical knowledge and clinical practice, which could bestow greater awareness of the content of our experiment.

The procedure was as follows. Subjects were randomly allocated to different computers, at which point they were given sufficient time to read the experimental instructions and sign informed consent forms. They were informed that the patient benefit in the experiment would be donated to help real patients. The participants were not allowed to communicate with each other; if they had questions, they could raise their hands, and the investigator would answer to them in private. Subject participation was predicted by answering a set of control questions and successfully completing a pilot experiment (see Additional File 2). In each part of the experiment, the subjects were assigned a rating of q for nine types of patients based on the information presented on the computer screen. The patients’ order was randomly determined during the experimental design and kept constant for all subjects under all experimental conditions. After the participants completed the decision-making tasks, they were asked to fill out a questionnaire covering areas such as the reason for participating in the experiment, determinants of decision-making, and overall feelings about the experiment (see Additional File 3).

Each group of experiments was conducted for five rounds, and 20,250 experimental records were collected. Talers (i.e., tokens) were used as the experimental currency; 1 Taler = 0.04 CNY. Subjects received a sum of *π*(*q*) for five rounds plus a basic reward of 30 CNY for their participation in the experiments; each subject earned 104 CNY on average. The sum of *B*(*q*) for one of the five rounds (chosen at random) was donated to the Red Cross Society of China. To ensure the donation’s authenticity, a subject was randomly selected as the monitor. After the experiment, the monitor verified that 2,158 CNY were transferred to the Red Cross Society of China through the financial department of the Capital Medical University. The monitor earned an additional 50 CNY as compensation for fulfilling this responsibility.

### Statistical analyses

The distribution of age, gender, and education among the five groups was not significantly different (*p* > 0.05). We analyzed the differences in physicians’ behavior and patient benefit using the (nonparametric) Friedman test. Dunn-Bonferroni correction was used for all post-hoc tests. All tests were two-sided, and the significance level was set at 0.05.

The fixed effects model (FEM) was used to test the robustness of the effects of different salary incentives and other control conditions on physicians’ behavior and patient benefit. Model **1 **is $Y_{itQdis}\;=\;\beta_1Salary_{it}+\beta_2k_{it}+\beta_3j_{it}+\lambda Z_i+u_i+\varepsilon_{it}$, where Q_*dis*_ is the deviation of quantity, which is calculated as ${Q}_{dis} = \left|q -{q}^{*}\right|$. Model **2** is $Y_{itLkj}\;=\;\beta_1Salary_{it}+\beta_2k_{it}+\beta_3j_{it}+\lambda Z_i+u_i+\varepsilon_{it}$, where *L*_*kj*_ is the loss ratio of the patient benefit calculated as follows: $L_{kj}=\lbrack B(q^\ast)-B(q)\rbrack/B(q^\ast)$. Salary is a set of dummy variables for different salary incentives in each group; k and j are the types and severity of illness; *Z*_*i*_ is a vector of individual characteristics; ${\text{u}_\text{i}}$ is an individual-specific effect that does not vary over time, and ε_*it*_ is an error term.

Pooled regression (PR) was used to test for differences in patient benefit among several salary incentives. The model is $Y_{itBenefit}\;=\;\beta_1Wage_{it}+\beta_2k_{it}+\beta_3j_{it}+\lambda{\mathrm Z}_{\mathrm i}+\mathrm U+{\mathrm\varepsilon}_{\mathrm{it}}$, where “Benefit” is the patient benefit in a certain part of each group; wage is a set of dummy variables for different types of salary incentives based on the fixed-wage group; k and j represent illness types and severity; *Z*_*i*_ is an individual characteristic variable; $U$ represents the same individual intercept, and ε_*it*_ is an error term.

Robust standard errors are clustered at the individual subject level in the regression model.

## Results

### Comparison of physicians’ behavior

#### Physicians’ behavior under fixed wage

In group I, the average quantities of medical service under t, 2t, and 3t were 4.71, 4.73, and 4.73, respectively (Table 2). The differences were statistically significant at the overall level (*p* = 0.005, Friedman test), but none were statistically significant after further pairwise comparisons. Regarding illness severity, although the differences in the quantity of services under different fixed wage levels were significant in moderate and severe severity (mild, *p* = 0.057 > 0.05; moderate, *p* = 0.008; severe, *p* = 0.009; Friedman test), none of the differences were significant after further pairwise comparisons.

**Table 2:** Average quantity of services under fixed wage

Salary Incentives	Overall		Mild		Moderate		Severe	
	Mean	SD	Mean	SD	Mean	SD	Mean	SD
t	4.71	2.08	2.89	1.40	4.66	1.39	6.58	1.49
2t	4.73	2.06	2.93	1.44	4.70	1.33	6.56	1.50
3t	4.73	2.04	2.89	1.32	4.68	1.28	6.61	1.45

Table 2 shows the average quantity of services under fixed wages in group I. t, 2t, and 3t refer to three levels of fixed wage. “Overall” refers to aggregate data for nine types of patients

#### Physicians’ behavior under floating wage level

**Fig. 1:**
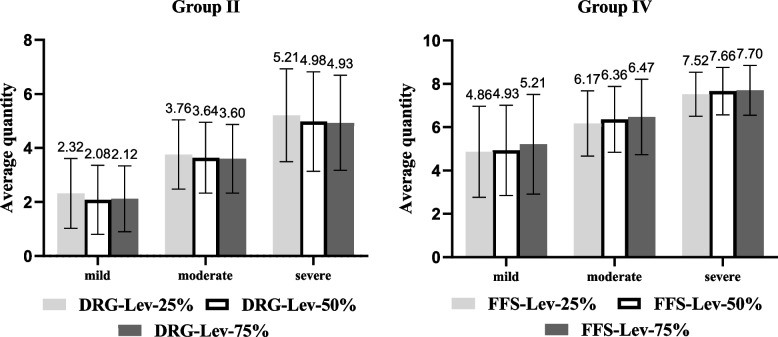
Average Quantity in Different Severities Under Various Salary Incentives in Group II, and IV

In group II, the average quantities of services under DRG-Lev-25%, DRG-Lev-50%, and DRG-Lev-75% were 3.76, 3.57, and 3.55, respectively (Table 3). The differences in physicians’ provision were statistically significant at the overall level (*p* < 0.001, Friedman test). Further pairwise comparisons revealed that physicians’ provisions decreased as the performance wage increased under DRG (DRG-Lev-25% vs. DRG-Lev-50%, adjusted *p* < 0.001; DRG-Lev-25% vs. DRG-Lev-75%, adjusted *p* < 0.001; DRG-Lev-50% vs. DRG-Lev-75%, adjusted *p* = 0.592 > 0.05). In terms of illness severity, the differences remained statistically significant (Fig. 1; mild, *p* < 0.001; moderate, *p* < 0.001; severe, *p* < 0.001; Friedman test), although it was slightly weaker between DRG-Lev-50% and DRG-Lev-75% in mild severity.

**Table 3:** Average quantity of services under different salary incentives based on DRG/FFS

Group	Salary Incentives	Mean	SD
II	DRG-Lev-25%	3.76	1.86
	DRG-Lev-50%	3.57	1.91
	DRG-Lev-75%	3.55	1.84
III	DRG-Com-25%	3.60	2.04
	DRG-Com-50%	3.59	2.07
	DRG-Com-75%	3.58	2.04
IV	FFS-Lev-25%	6.18	1.94
	FFS-Lev-50%	6.32	1.96
	FFS-Lev-75%	6.46	2.06
V	FFS-Com-25%	5.95	1.89
	FFS-Com-50%	5.96	1.88
	FFS-Com-75%	6.03	1.86

Table 3 shows the average quantity of services under different salary incentives based on DRG/FFS. DRG (FFS)-Lev-25%, DRG (FFS)-Lev-50%, DRG (FFS)-Lev-75% refer to different levels of floating wage system that consists of constant fixed wage and variable performance wage in group II (IV). DRG (FFS)-Com-25%, DRG (FFS)-Com-50%, DRG (FFS)-Com-75% refer to different compositions of floating wage system that consists of variable fixed wage and variable performance wage in group III (V)

In group IV, the average quantities of services under FFS-Lev-25%, FFS-Lev-50%, and FFS-Lev-75% were 6.18, 6.32, and 6.46, respectively (Table 3). The differences in physicians’ provision were statistically significant at the overall level (*p* < 0.001, Friedman test) and remained significant after further pairwise comparisons (FFS-Lev-25% vs. FFS-Lev-50%, adjusted *p* = 0.001; FFS-Lev-25% vs. FFS-Lev-75%, adjusted *p* < 0.001; and FFS-Lev-50% vs. FFS-Lev-75%, adjusted *p* = 0.008). Regarding illness severity, differences in the quantity of services under different wage levels were also significant (Fig. 1; mild, *p* < 0.001; moderate, *p* < 0.001; severe, *p* < 0.001; Friedman test). This indicates that the physicians’ provision increased as the performance wage increased under FFS.

#### Physicians’ behavior under floating wage composition

**Fig. 2:**
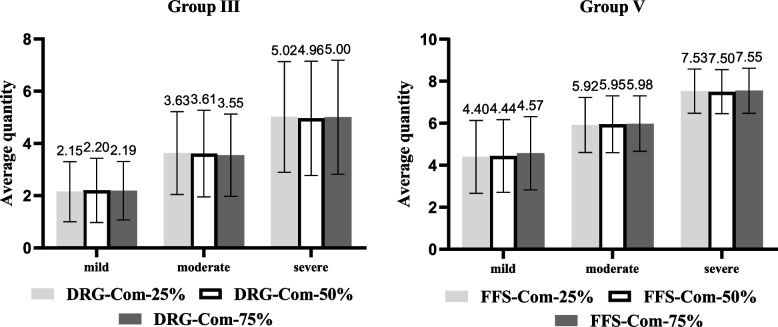
Average Quantity in Different Severities Under Various Salary Incentives in Group III, and V

In group III, the average quantities of services under DRG-Com-25%, DRG-Com-50%, and DRG-Com-75% were 3.60, 3.59, and 3.58, respectively (Table 3). The differences were not statistically significant at the overall level (*p* = 0.166 > 0.05, Friedman test) and by illness severity (mild, *p* = 0.800 > 0.05; moderate, *p* = 0.002, with all adjusted *p* > 0.05 after further pairwise comparisons; severe, *p* = 0.862 > 0.05, Friedman test). We observed no significant difference in physicians’ provision under DRG-Com-25%, DRG-Com-50%, and DRG-Com-75% (Fig. 2).

In group V, the average quantities of services under FFS-Com-25%, FFS-Com-50%, and FFS-Com-75% were 5.95, 5.96, and 6.03, respectively (Table 3). The differences were statistically significant at the overall level (*p* < 0.001, Friedman test). After further pairwise comparisons, the differences were statistically significant, except for FFS-Com-25% vs. FFS-Com-50%. This indicates that, compared to FFS-Com-75%, the physicians’ provision was lower under FFS-Com-25% and FFS-Com-50%. In terms of illness severity, the difference was statistically significant only in FFS-Com-25% vs. FFS-Com-75% (adjusted *p* = 0.004; Friedman test) for mild severity (Fig. 2).

### Comparison of patient benefit

#### Patient benefit under fixed wage

The average patient benefits under t, 2t, and 3t were 14.08, 14.11, and 14.13, respectively (Table 4). The differences were statistically significant at the overall level (*p* = 0.005, Friedman test), but after further pairwise comparisons, the differences between t, 2t, and 3t were not statistically significant (t vs. 2t, t vs. 3t, and 2t vs. 3t, all adjusted *p* > 0.05). When considering illness severity, we observed similar results (mild, *p* = 0.760 > 0.05; moderate, *p* = 0.011; severe, *p* = 0.056 > 0.05). However, we found no significant differences after further pairwise comparisons.

**Table 4:** Average health benefit in each group

Group	Salary Incentives	Overall		Mild		Moderate		Severe	
		Mean	SD	Mean	SD	Mean	SD	Mean	SD
I	t	14.08	4.23	14.20	4.25	14.06	4.20	14.00	4.22
	2t	14.11	4.22	14.20	4.26	14.11	4.16	14.00	4.24
	3t	14.13	4.20	14.25	4.25	14.12	4.13	14.03	4.21
II	DRG-Lev-25%	13.20	4.06	13.75	4.00	13.25	3.95	12.58	4.14
	DRG-Lev-50%	12.96	4.00	13.56	3.98	13.04	3.73	12.30	4.17
	DRG-Lev-75%	12.92	3.99	13.54	3.91	13.02	3.85	12.21	4.10
III	DRG-Com-25%	12.99	4.21	13.64	3.98	13.01	4.09	12.33	4.43
	DRG-Com-50%	12.94	4.22	13.64	3.99	12.97	4.11	12.20	4.41
	DRG-Com-75%	12.96	4.20	13.68	3.96	12.95	4.08	12.24	4.42
IV	FFS-Lev-25%	13.27	4.18	12.60	4.35	13.37	4.07	13.85	4.02
	FFS-Lev-50%	13.08	4.07	12.48	4.26	13.00	3.96	13.78	3.89
	FFS-Lev-75%	12.87	4.17	12.18	4.44	12.82	3.99	13.60	3.93
V	FFS-Com-25%	13.53	4.05	12.93	4.07	13.54	3.94	14.13	4.05
	FFS-Com-50%	13.49	4.07	12.85	4.04	13.50	4.02	14.13	4.04
	FFS-Com-75%	13.45	4.07	12.74	4.07	13.53	4.03	14.08	3.99

Table 4 shows the average health benefits in each group. t, 2t, 3t refer to three levels of fixed wage. DRG (FFS)-Lev-25%, DRG (FFS)-Lev-50%, DRG (FFS)-Lev-75% refer to different levels of floating wage system that consists of constant fixed wage and variable performance wage in group II (IV). DRG (FFS)-Com-25%, DRG (FFS)-Com-50%, DRG (FFS)-Com-75% refer to different compositions of floating wage system that consists of variable fixed wage and variable performance wage in group III (V). “Overall” refers to aggregate data for nine types of patients

#### Patient benefit under floating wage level

In group II, the average patient benefits under DRG-Lev-25%, DRG-Lev-50%, and DRG-Lev-75% were 13.20, 12.96, and 12.92, respectively (Table 4). The differences were statistically significant at the overall level (*p* < 0.001, Friedman test) and remained significant after further pairwise comparisons, except for DRG-Lev-50% vs. DRG-Lev-75% (adjusted *p* = 0.203 > 0.05). In terms of illness severity, the differences in patient benefit were also statistically significant (mild, *p* < 0.001; moderate, *p* < 0.001; severe, *p* < 0.001; Friedman test). This suggests that patient benefit decreased as the performance wage increased under DRG.

In group IV, the average patient benefits under FFS-Lev-25%, FFS-Lev-50%, and FFS-Lev-75% were 13.27, 13.08, and 12.87, respectively (Table 4). The differences were statistically significant at the overall level (*p* < 0.001 and all adjusted *p* < 0.05 after further pairwise comparisons, Friedman test). In terms of illness severity, the differences in patient benefit at different salary levels were statistically significant (mild, *p* < 0.001; moderate, *p* < 0.001; severe, *p* < 0.001; Friedman test). The results showed that patient benefit decreased with the increase of performance wage under FFS.

#### Patient benefit under floating wage composition

In group III, the average patient benefits under DRG-Com-25%, DRG-Com-50%, and DRG-Com-75% were 12.99, 12.94, and 12.96, respectively (Table 4). The differences were not statistically significant at the overall level (*p* = 0.231 > 0.05, Friedman test) or by illness severity (mild, *p* = 0.501 > 0.05; moderate, *p* = 0.058 > 0.05; severe, *p* = 0.207 > 0.05; Friedman test), indicating no significant difference in patient benefits under DRG-Com-25%, DRG-Com-50%, and DRG-Com-75%.

In group V, the average patient benefits with FFS-Com-25%, FFS-Com-50%, and FFS-Com-75% were 13.53, 13.49, and 13.45, respectively (Table 4). The differences were statistically significant at the overall level (*p* = 0.002, Friedman test) but not significant after further pairwise comparisons. In terms of illness severity, the difference under different wage compositions was significant only for severity mild (mild, *p* < 0.001; moderate, *p* = 0.221 > 0.05; severe, *p* = 0.498 > 0.05; Friedman test). After further pairwise comparisons, we found that compared to FFS-Com-25%, patient benefits decreased with a higher ratio of performance wage under FFS-Com-75% for severity mild (FFS-Com-25% vs. FFS-Com-75%, adjusted *p* = 0.026).

### Regression analysis of deviation of quantity and patient benefit

The Panel effect model was used to analyze the deviation of quantity and loss ratio of patient benefit under different salary incentives in each group. Table 5 summarizes the FEM regression results. The dependent variable in Panel A was *Q*_*dis*_, and that in Panel B was *L*_*kj*_. The results indicated no significant difference in deviation of quantity and loss ratio of patient benefit under different levels of fixed wage. In groups II and IV, where the floating wage levels were examined, the results showed that the deviation of quantity and loss ratio of patient benefit increased as the performance wage based on the payment method balance increased. This effect was particularly pronounced in the comparison between DRG-Lev-25% and DRG-Lev-75% as well as between FFS-Lev-25% and FFS-Lev-75%. However, we found no significant differences in the deviation of quantity and loss ratio of patient benefit under different floating wage compositions in groups III and V.

**Table 5:** FEM regression of deviation of quantity and loss ratio of patient benefit in each group

Independent variable	${\boldsymbol Q}_{\mathbf d\mathbf i\mathbf s}$ (Panel A)					${\boldsymbol L}_{\mathbf k\mathbf j}$ (Panel B)				
	(1)	(2)	(3)	(4)	(5)	(6)	(7)	(8)	(9)	(10)
**Payment: t/DRG(FFS)-Lev-25% / DRG(FFS)-Com-25% (ref)**										
2t/ DRG(FFS)-Lev-50% / DRG(FFS)-Com-50%	− 0.019	0.168**	0.039	0.120	0.031	− 0.002	0.014**	0.003	0.010	0.003
	(0.014)	(0.058)	(0.040)	(0.068)	(0.027)	(0.001)	(0.005)	(0.004)	(0.006)	(0.002)
3t/ DRG(FFS)-Lev-75% / DRG(FFS)-Com-75%	− 0.041	0.194**	0.018	0.288**	0.058	− 0.003	0.018**	0.002	0.024**	0.006
	(0.022)	(0.070)	(0.042)	(0.096)	(0.041)	(0.002)	(0.006)	(0.004)	(0.009)	(0.004)
**Type of illness: illness A (ref)**										
Illness B	− 0.021	0.210*	0.118	0.220*	0.161	− 0.024**	− 0.032***	− 0.042***	− 0.017	− 0.024**
	(0.065)	(0.083)	(0.077)	(0.087)	(0.091)	(0.008)	(0.007)	(0.010)	(0.014)	(0.007)
Illness C	0.015	0.115	− 0.015	0.193	0.106	0.001	0.011	− 0.001	0.041	0.011
	(0.058)	(0.089)	(0.109)	(0.112)	(0.090)	(0.006)	(0.009)	(0.011)	(0.024)	(0.009)
**Severity of illness: Mild (ref)**										
Moderate	0.090	0.373**	0.511**	− 0.627***	− 0.515***	0.008	0.033**	0.045**	− 0.042*	− 0.045***
	(0.125)	(0.122)	(0.144)	(0.147)	(0.102)	(0.011)	(0.011)	(0.013)	(0.016)	(0.009)
Severe	0.157	0.936**	1.047***	− 1.227***	− 0.959***	0.013	0.083**	0.092***	− 0.087**	− 0.084***
	(0.207)	(0.274)	(0.272)	(0.280)	(0.164)	(0.018)	(0.024)	(0.024)	(0.030)	(0.014)
Constant	0.604***	0.812***	0.967***	1.789***	1.499***	0.061***	0.087***	0.103***	0.147***	0.144***
	(0.123)	(0.159)	(0.164)	(0.155)	(0.089)	(0.011)	(0.013)	(0.013)	(0.024)	(0.009)
Observations	4050	4050	4050	4050	4050	4050	4050	4050	4050	4050
Subjects	30	30	30	30	30	30	30	30	30	30
R^2^	0.011	0.123	0.167	0.215	0.230	0.042	0.138	0.184	0.132	0.246

This table shows results from the fixed effects model. The dependent variable is the deviation of quantity (Panel A, columns 1 to 5) and loss ratio of patient benefit (Panel B, columns 6 to 10). Columns 1, 6; 2, 7; 3, 8; 4, 9; and 5,10 show regression results for groups I, II, III, IV, and V, respectively. The reference category is t in group I; DRG (FFS)-Lev-25% in group II (IV); DRG (FFS)-Com-25% in group III (V). Additionally, we control for the type of illness and severity with illness ‘A’ and severity ‘mild’ being the reference categories. Robust standard errors, in parentheses below the coefficients, are clustered at individual subjects

^*^ *p* < 0.05

^**^ *p* < 0.01

^***^ *p* < 0.001

Since we observed no significant differences in patient benefit among the three parts in group I, we aggregated the data of patient benefit at different levels of fixed wage to compare patient benefit for different types of salary incentives. Additionally, considering that patient benefit was the highest under the salary incentives of DRG-Lev-25%, FFS-Lev-25%, DRG-Com-25%, and FFS-Com-25% in groups II, III, IV, and V, respectively, we compared the patient benefit of those parts with fixed wages to examine which type of salary incentive could yield the highest patient benefit. As shown in Table 6, the results demonstrated that patient benefit was highest under fixed wages, even after controlling for other variables such as k and j.

**Table 6:** Pooled regression of patient benefit among different types of salary incentives

Independent variable	Benefit		
	(1)	(2)	(3)
**Payment: fixed wages (ref)**			
DRG-Lev-25%	-0.912**	-0.912**	-0.916**
	(0.344)	(0.344)	(0.328)
DRG-Com-25%	-1.115**	-1.115**	-1.106**
	(0.409)	(0.409)	(0.411)
FFS-Lev-25%	-0.835*	-0.835*	-0.851*
	(0.390)	(0.390)	(0.381)
FFS-Com-25%	-0.576	-0.576	-0.586
	(0.374)	(0.374)	(0.368)
**Type of illness: illness A (ref)**			
Illness B		4.910***	4.910***
		(0.040)	(0.040)
Illness C		8.888***	8.888***
		(0.139)	(0.139)
**Severity of illness: Mild (ref)**			
Moderate		-0.012	-0.012
		(0.095)	(0.095)
Severe		-0.092	-0.092
		(0.170)	(0.170)
Age			0.005
			(0.097)
Gender (female for ref)			0.133
			(0.353)
Education (undergraduates for ref)			-0.351
			(0.482)
Constant	14.108***	9.544***	9.557***
	(0.268)	(0.251)	(2.097)
Observations	9450	9450	9450
Subjects	150	150	150
R^2^	0.012	0.765	0.767

This table shows results from the pooled regression. The dependent variable is the patient benefit under fixed wage, DRG (FFS)-Lev-25%, DRG (FFS)-Com-25%. The reference category is fixed wages in group I. Also, we control for the type of illness and severity with illness ‘A’ and severity ‘mild’ being the reference categories. The demographics of variables comprise age, gender, and education. Robust standard errors, in parentheses below the coefficients, are clustered at individual subjects

^*^ *p* < 0.05

^**^ *p* < 0.01

^***^ *p* < 0.001

## Discussion

Under the framework of an indirect incentive path of payment methods, we conducted a laboratory experiment to examine the effects of internal salary incentives on physicians’ behavior. Our study yielded three main findings. First, our results indicated that a moderate change in fixed wage levels did not have a significant impact on physicians’ behavior. Previous experiments using a gift-exchange game have demonstrated that as participants’ fixed wages increased, their effort levels also increased as positive reciprocal subjects responded to the generous fixed wage with higher effort levels [29, 45, 46]. Manthei and Mohnen [30] found that individuals with either low or high fixed wages worked more than those with intermediate fixed wages. The lower effort levels observed for subjects with intermediate fixed wages compared to those with high fixed wages could be interpreted as a social norm, where a certain level of effort should correspond to a certain fixed wage. In our experiment, we employed a different design from that of Manthei and Mohnen [30] as we varied the level of fixed wages within a pure fixed wage system; conversely, Manthei and Mohnen [30] used a linear incentive contract that varied the fixed wage but kept the piece rate constant across treatments. In our study, although the level of fixed wages differed among the three parts in group I, physicians’ salaries remained constant regardless of the quantity of medical services provided in each part. In this case, physicians preferred to choose the optimal quantity that benefited patients the most, resulting in no significant differences in the physicians’ quantity choice and patient benefit between the three parts in group I. Furthermore, in our experiment, the highest fixed wage was only twice as much as the lowest fixed wage, which was not considered very high. Considering previous experimental results [29, 45, 46], we believe that a higher level of fixed wage may lead to a significant alteration in physicians’ behavior.

Second, we found that under the floating wage system, which consisted of constant fixed wage and variable performance wage, physicians’ quantity choices decreased (increased) with an increase in the performance wage under the DRG (FFS) payment. This finding is consistent with the predictions of the simple principal-agent model, which suggests that agent performance increases with stronger incentives, ceteris paribus [30]. It suggests that the incentive effect of payment methods can be indirectly transmitted to physicians using the payment method balance to issue physician performance wages. However, it is essential to examine what factors lead to the increase in performance wage and how the physicians’ behavior changes. Based on the Holmstrom-Milgrom (HM) model [47], if the principal imposes a strong incentive contract, the agent will allocate more effort to tasks with high performance measurability and less effort to tasks with lower performance measurability. In the experiments conducted by Brosig-Koch et al. [16, 17] and Oxholm, Guida, and Gyrd-Hansen [33], the P4P bonus was only awarded to physicians when their quantity choice reached a quality threshold related to maximum patient benefit. Therefore, P4P incentivizes physicians to provide the optimal quantity, which not only improves patient benefit but also increases the physicians’ own profits. In contrast to their experimental design, in groups II and IV of our experiment, the increase in physicians’ salaries came from the payment method balance. As the quantity choices of physicians under DRG (FFS) approached 0 (10), the payment method balance increased, leading to higher salaries for physicians. Consequently, to obtain higher personal profits, the quantity provided by physicians deviated more from the optimal quantity, resulting in a lower patient benefit. This reminds us that while performance wages have a substantial impact on personal motivation, the crucial factor lies in establishing appropriate measurement standards for the allocation of such remuneration.

Third, our results suggested that under the condition of unchanged salary level but changes in salary composition, physicians’ behavior showed slight changes under FFS, especially in the comparison between FFS-Com-25% and FFS-Com-75%. However, we observed no significant change in physicians’ behavior under DRG. This indicates that salary composition may affect physicians’ behavior in some situations. Among the three different salary compositions, the proportion of performance wages in the total salary was higher under FFS (approximately 11.69% in group V and 10.59% in group III) than under DRG, and the magnitude of variation in performance wages under FFS was also greater, which may explain why the changes in physicians’ behavior were more pronounced under FFS payment. The comparison between the results of Gneezy and Rustichini [48], and Pokorny [49] also suggests that salary composition may affect the effectiveness of incentives. It would be useful to conduct further research to examine how changes in the ratios between fixed wage and performance wage influence subjects’ behavior; this will help clarify which composition of fixed wage and performance wage is the most appropriate incentive approach.

Besides, our results demonstrated that physicians did not always select the quantity that would maximize their personal profits. This finding suggests that physicians possess altruistic preferences, prioritizing health benefit over their financial gain. As mentioned in existing literature, physicians’ behavior may be influenced by social preferences such as altruism and moral norms, which possibly play a significant role in shaping their behavior [50]. However, Müller, Schmid, and Gerfin [51] found that despite physicians’ reluctance to prioritize profit over patient benefit, they still exhibited rent-seeking behavior, resulting in inefficient utilization of resources and unnecessary costs in the healthcare system.

Our findings have meaningful policy implications. The multitasking problem [47, 52] suggests that explicitly rewarding providers for specific aspects might cause them to focus more on tasks that are measured and rewarded, while neglecting non-incentivized tasks. Cattel et al. [53] propose a value-based provider payment consisting of two components: a relatively large base payment that implicitly stimulates key-value and a relatively small payment (pay-for-performance) that explicitly rewards measurable aspects of value to avoid the risk of multitasking and promote value in healthcare. The results in Table 6 showed that patient benefit was highest under fixed wages, which indicates that physicians may be more patient-oriented under fixed wages than under other types of compensation. Based on our experimental results, linking physicians’ performance wage with payment method balance may be effective in transferring the incentive effect of payment methods to physicians. However, in the long term, the proportion of fixed wage should be gradually increased considering the advantages of fixed wage in guaranteeing quality. Wu and Li [36] suggest that physicians’ salaries should mainly consist of a fixed wage, with the proportion of performance wage to total salary not exceeding 20%. Additionally, it is essential to exercise caution when utilizing performance wage as it may lead to the crowding out of motivation [16, 32, 54]. In particular, in cases where medical quality is not or partially included in the measurement of physician performance, or when the performance measurement criteria are imperfect [52].

Our study has certain limitations as our theoretical framework and experimental setting do not encompass all the factors that may influence physicians’ behavior. We assumed that physicians’ performance wage came solely from payment method balance. In reality, the calculation and distribution process of physicians’ performance wage is complicated and involves many interfering factors. In addition, the abstraction of the experimental parameters weakened the external validity of the experimental results. Moreover, the design of “patient benefit” is evident and quantified. Actually, it is challenging for physicians to quantify or determine specific patient benefits based on the services they provide. Additionally, the choice of subject pool has been shown to be an issue in investigating participant behaviors. Previous experimental evidence [1, 32, 55] has shown that physicians’ responses to different compensation schemes are qualitatively similar to medical students, but medical students are less patient-oriented. Brosig-Koch et al. [16] found that physicians are more sensitive to P4P incentives compared to medical students. Undoubtedly, the external validity of laboratory experiments constitutes an issue; however, we believe that the findings obtained in a controlled environment can reflect physicians’ underlying decision-making mechanisms and contribute to exploring the interactions between service providers, patients, and different financial incentives.

## Conclusions

Based on the indirect incentive path of payment methods, utilizing payment method balance for physicians’ salaries can be effective in transferring the incentive effect of payment methods to physicians. However, the increase in salary levels resulting from an increase in the distribution of payment method balance led to a decrease in patient benefit. This suggests that this type of physician compensation design should be approached with caution, especially when the measurement standard for physicians’ performance is flawed. Further comprehensive studies are required to optimize the design of internal salary incentives to effectively transmit the incentive effect of payment methods to physicians while ensuring medical quality. Taking into account the relative advantages of fixed wages in guaranteeing quality of care, we suggest that the physician salary system incorporate a combination of fixed wage and performance wage that is contingent upon value assessment.

### Supplementary Information

**Additional file 1.** Parameter Tables.

**Additional file 2.** Instructions + Comprehension Questions.

**Additional file 3.** Questionnaire Survey.

## Data Availability

The datasets generated and/or analyzed during the current study are not publicly available due to ethical issues but are available from the corresponding author on reasonable request.

## Abbreviations

FFS: Fee-for-service
CAP: Capitation
P4P: Pay-for-performance
DRG: Diagnosis-related-group
DRG-Lev-25%, DRG-Lev-50%, DRG-Lev-75%: Fixed wages are constant and performance wages are 25%, 50% and 75% of the payment method balance under DRG in group II
FFS-Lev-25%, FFS-Lev-50%, FFS-Lev-75%: Fixed wages are constant and performance wages are 25%, 50% and 75% of the payment method balance under FFS in group IV
DRG-Com-25%, DRG-Com-50%, DRG-Com-75%: Fixed wages are variable and performance wages are 25%, 50% and 75% of the payment method balance under DRG in group III
FFS-Com-25%, FFS-Com-50%, FFS-Com-75%: Fixed wages are variable and performance wages are 25%, 50% and 75% of the payment method balance under FFS in group V
SD: Standard deviation
FEM: Fixed effects model
PR: Pooled regression
