# Supplementary material for: The effect of internal salary incentives based on insurance payment on physicians’ behavior: experimental evidence
Source: BMC Health Serv Res. 2023 Dec 14;23:1410. doi: 10.1186/s12913-023-10408-8 (PMC10720113; doi:10.1186/s12913-023-10408-8)
Supplement: Supplementary file 1 — Additional file 1. Parameter Tables. [file 12913_2023_10408_MOESM1_ESM.docx]

**Additional File 1: Parameter Tables**

Table S1 Experimental Parameters of Profit in Group II (Under DRG)

| **Quantity (q)** | | **0** | **1** | **2** | **3** | **4** | **5** | **6** | **7** | **8** | **9** | **10** |
| --- | --- | --- | --- | --- | --- | --- | --- | --- | --- | --- | --- | --- |
| DRG-Lev-25% | A_l_ | 9.43 | 9.41 | 9.33 | 9.21 | 9.03 | 8.81 | 8.53 | 8.21 | 7.83 | 7.41 | 6.93 |
|  | A_m_ | 10.39 | 10.36 | 10.29 | 10.16 | 9.99 | 9.76 | 9.49 | 9.16 | 8.79 | 8.36 | 7.89 |
|  | A_h_ | 11.34 | 11.32 | 11.24 | 11.12 | 10.94 | 10.72 | 10.44 | 10.12 | 9.74 | 9.32 | 8.84 |
|  | B_l_ | 9.50 | 9.48 | 9.40 | 9.28 | 9.10 | 8.88 | 8.60 | 8.28 | 7.90 | 7.48 | 7.00 |
|  | B_m_ | 10.50 | 10.48 | 10.40 | 10.28 | 10.10 | 9.88 | 9.60 | 9.28 | 8.90 | 8.48 | 8.00 |
|  | B_h_ | 11.50 | 11.48 | 11.40 | 11.28 | 11.10 | 10.88 | 10.60 | 10.28 | 9.90 | 9.48 | 9.00 |
|  | C_l_ | 9.58 | 9.55 | 9.48 | 9.35 | 9.18 | 8.95 | 8.68 | 8.35 | 7.98 | 7.55 | 7.08 |
|  | C_m_ | 10.63 | 10.60 | 10.53 | 10.40 | 10.23 | 10.00 | 9.73 | 9.40 | 9.03 | 8.60 | 8.13 |
|  | C_h_ | 11.68 | 11.65 | 11.58 | 11.45 | 11.28 | 11.05 | 10.78 | 10.45 | 10.08 | 9.65 | 9.18 |
| DRG-Lev-50% | A_l_ | 10.87 | 10.82 | 10.67 | 10.42 | 10.07 | 9.62 | 9.07 | 8.42 | 7.67 | 6.82 | 5.87 |
|  | A_m_ | 12.78 | 12.73 | 12.58 | 12.33 | 11.98 | 11.53 | 10.98 | 10.33 | 9.58 | 8.73 | 7.78 |
|  | A_h_ | 14.69 | 14.64 | 14.49 | 14.24 | 13.89 | 13.44 | 12.89 | 12.24 | 11.49 | 10.64 | 9.69 |
|  | B_l_ | 11.00 | 10.95 | 10.80 | 10.55 | 10.20 | 9.75 | 9.20 | 8.55 | 7.80 | 6.95 | 6.00 |
|  | B_m_ | 13.00 | 12.95 | 12.80 | 12.55 | 12.20 | 11.75 | 11.20 | 10.55 | 9.80 | 8.95 | 8.00 |
|  | B_h_ | 15.00 | 14.95 | 14.80 | 14.55 | 14.20 | 13.75 | 13.20 | 12.55 | 11.80 | 10.95 | 10.00 |
|  | C_l_ | 11.15 | 11.10 | 10.95 | 10.70 | 10.35 | 9.90 | 9.35 | 8.70 | 7.95 | 7.10 | 6.15 |
|  | C_m_ | 13.25 | 13.20 | 13.05 | 12.80 | 12.45 | 12.00 | 11.45 | 10.80 | 10.05 | 9.20 | 8.25 |
|  | C_h_ | 15.35 | 15.30 | 15.15 | 14.90 | 14.55 | 14.10 | 13.55 | 12.90 | 12.15 | 11.30 | 10.35 |
| DRG-Lev-75% | A_l_ | 12.30 | 12.22 | 12.00 | 11.62 | 11.10 | 10.42 | 9.60 | 8.62 | 7.50 | 6.22 | 4.80 |
|  | A_m_ | 15.16 | 15.09 | 14.86 | 14.49 | 13.96 | 13.29 | 12.46 | 11.49 | 10.36 | 9.09 | 7.66 |
|  | A_h_ | 18.03 | 17.95 | 17.73 | 17.35 | 16.83 | 16.15 | 15.33 | 14.35 | 13.23 | 11.95 | 10.53 |
|  | B_l_ | 12.50 | 12.43 | 12.20 | 11.83 | 11.30 | 10.63 | 9.80 | 8.83 | 7.70 | 6.43 | 5.00 |
|  | B_m_ | 15.50 | 15.43 | 15.20 | 14.83 | 14.30 | 13.63 | 12.80 | 11.83 | 10.70 | 9.43 | 8.00 |
|  | B_h_ | 18.50 | 18.43 | 18.20 | 17.83 | 17.30 | 16.63 | 15.80 | 14.83 | 13.70 | 12.43 | 11.00 |
|  | C_l_ | 12.73 | 12.65 | 12.43 | 12.05 | 11.53 | 10.85 | 10.03 | 9.05 | 7.93 | 6.65 | 5.23 |
|  | C_m_ | 15.88 | 15.80 | 15.58 | 15.20 | 14.68 | 14.00 | 13.18 | 12.20 | 11.08 | 9.80 | 8.38 |
|  | C_h_ | 19.03 | 18.95 | 18.73 | 18.35 | 17.83 | 17.15 | 16.33 | 15.35 | 14.23 | 12.95 | 11.53 |

Table S2 Experimental Parameters of Profit in Group III (Under DRG)

| **Quantity (q)** | | | **0** | **1** | **2** | **3** | **4** | **5** | **6** | **7** | **8** | **9** | **10** |
| --- | --- | --- | --- | --- | --- | --- | --- | --- | --- | --- | --- | --- | --- |
| DRG-  Com  -25% | Fixed  wage | A_l_ | 10.87 | 10.82 | 10.67 | 10.42 | 10.07 | 9.62 | 9.07 | 8.42 | 7.67 | 6.82 | 5.87 |
|  |  | A_m_ | 12.78 | 12.73 | 12.58 | 12.33 | 11.98 | 11.53 | 10.98 | 10.33 | 9.58 | 8.73 | 7.78 |
|  |  | A_h_ | 14.69 | 14.64 | 14.49 | 14.24 | 13.89 | 13.44 | 12.89 | 12.24 | 11.49 | 10.64 | 9.69 |
|  |  | B_l_ | 11.00 | 10.95 | 10.80 | 10.55 | 10.20 | 9.75 | 9.20 | 8.55 | 7.80 | 6.95 | 6.00 |
|  |  | B_m_ | 13.00 | 12.95 | 12.80 | 12.55 | 12.20 | 11.75 | 11.20 | 10.55 | 9.80 | 8.95 | 8.00 |
|  |  | B_h_ | 15.00 | 14.95 | 14.80 | 14.55 | 14.20 | 13.75 | 13.20 | 12.55 | 11.80 | 10.95 | 10.00 |
|  |  | C_l_ | 11.15 | 11.10 | 10.95 | 10.70 | 10.35 | 9.90 | 9.35 | 8.70 | 7.95 | 7.10 | 6.15 |
|  |  | C_m_ | 13.25 | 13.20 | 13.05 | 12.80 | 12.45 | 12.00 | 11.45 | 10.80 | 10.05 | 9.20 | 8.25 |
|  |  | C_h_ | 15.35 | 15.30 | 15.15 | 14.90 | 14.55 | 14.10 | 13.55 | 12.90 | 12.15 | 11.30 | 10.35 |
|  | Payment  Method  Balance | A_l_ | 1.43 | 1.41 | 1.33 | 1.21 | 1.03 | 0.81 | 0.53 | 0.21 | -0.17 | -0.59 | -1.07 |
|  |  | A_m_ | 2.39 | 2.36 | 2.29 | 2.16 | 1.99 | 1.76 | 1.49 | 1.16 | 0.79 | 0.36 | -0.11 |
|  |  | A_h_ | 3.34 | 3.32 | 3.24 | 3.12 | 2.94 | 2.72 | 2.44 | 2.12 | 1.74 | 1.32 | 0.84 |
|  |  | B_l_ | 1.50 | 1.48 | 1.40 | 1.28 | 1.10 | 0.88 | 0.60 | 0.28 | -0.10 | -0.53 | -1.00 |
|  |  | B_m_ | 2.50 | 2.48 | 2.40 | 2.28 | 2.10 | 1.88 | 1.60 | 1.28 | 0.90 | 0.48 | 0.00 |
|  |  | B_h_ | 3.50 | 3.48 | 3.40 | 3.28 | 3.10 | 2.88 | 2.60 | 2.28 | 1.90 | 1.48 | 1.00 |
|  |  | C_l_ | 1.58 | 1.55 | 1.48 | 1.35 | 1.18 | 0.95 | 0.68 | 0.35 | -0.03 | -0.45 | -0.93 |
|  |  | C_m_ | 2.63 | 2.60 | 2.53 | 2.40 | 2.23 | 2.00 | 1.73 | 1.40 | 1.03 | 0.60 | 0.13 |
|  |  | C_h_ | 3.68 | 3.65 | 3.58 | 3.45 | 3.28 | 3.05 | 2.78 | 2.45 | 2.08 | 1.65 | 1.18 |
| DRG-  Com  -50% | Fixed  wage | A_l_ | 9.43 | 9.41 | 9.33 | 9.21 | 9.03 | 8.81 | 8.53 | 8.21 | 7.83 | 7.41 | 6.93 |
|  |  | A_m_ | 10.39 | 10.36 | 10.29 | 10.16 | 9.99 | 9.76 | 9.49 | 9.16 | 8.79 | 8.36 | 7.89 |
|  |  | A_h_ | 11.34 | 11.32 | 11.24 | 11.12 | 10.94 | 10.72 | 10.44 | 10.12 | 9.74 | 9.32 | 8.84 |
|  |  | B_l_ | 9.50 | 9.48 | 9.40 | 9.28 | 9.10 | 8.88 | 8.60 | 8.28 | 7.90 | 7.48 | 7.00 |
|  |  | B_m_ | 10.50 | 10.48 | 10.40 | 10.28 | 10.10 | 9.88 | 9.60 | 9.28 | 8.90 | 8.48 | 8.00 |
|  |  | B_h_ | 11.50 | 11.48 | 11.40 | 11.28 | 11.10 | 10.88 | 10.60 | 10.28 | 9.90 | 9.48 | 9.00 |
|  |  | C_l_ | 9.58 | 9.55 | 9.48 | 9.35 | 9.18 | 8.95 | 8.68 | 8.35 | 7.98 | 7.55 | 7.08 |
|  |  | C_m_ | 10.63 | 10.60 | 10.53 | 10.40 | 10.23 | 10.00 | 9.73 | 9.40 | 9.03 | 8.60 | 8.13 |
|  |  | C_h_ | 11.68 | 11.65 | 11.58 | 11.45 | 11.28 | 11.05 | 10.78 | 10.45 | 10.08 | 9.65 | 9.18 |
|  | Payment  Method  Balance | A_l_ | 2.87 | 2.82 | 2.67 | 2.42 | 2.07 | 1.62 | 1.07 | 0.42 | -0.34 | -1.19 | -2.14 |
|  |  | A_m_ | 4.78 | 4.73 | 4.58 | 4.33 | 3.98 | 3.53 | 2.98 | 2.33 | 1.58 | 0.73 | -0.23 |
|  |  | A_h_ | 6.69 | 6.64 | 6.49 | 6.24 | 5.89 | 5.44 | 4.89 | 4.24 | 3.49 | 2.64 | 1.69 |
|  |  | B_l_ | 3.00 | 2.95 | 2.80 | 2.55 | 2.20 | 1.75 | 1.20 | 0.55 | -0.20 | -1.05 | -2.00 |
|  |  | B_m_ | 5.00 | 4.95 | 4.80 | 4.55 | 4.20 | 3.75 | 3.20 | 2.55 | 1.80 | 0.95 | 0.00 |
|  |  | B_h_ | 7.00 | 6.95 | 6.80 | 6.55 | 6.20 | 5.75 | 5.20 | 4.55 | 3.80 | 2.95 | 2.00 |
|  |  | C_l_ | 3.15 | 3.10 | 2.95 | 2.70 | 2.35 | 1.90 | 1.35 | 0.70 | -0.05 | -0.90 | -1.85 |
|  |  | C_m_ | 5.25 | 5.20 | 5.05 | 4.80 | 4.45 | 4.00 | 3.45 | 2.80 | 2.05 | 1.20 | 0.25 |
|  |  | C_h_ | 7.35 | 7.30 | 7.15 | 6.90 | 6.55 | 6.10 | 5.55 | 4.90 | 4.15 | 3.30 | 2.35 |
| DRG-  Com  -75% | Fixed  wage | A_l_ | 8.00 | 8.00 | 8.00 | 8.00 | 8.00 | 8.00 | 8.00 | 8.00 | 8.00 | 8.00 | 8.00 |
|  |  | A_m_ | 8.00 | 8.00 | 8.00 | 8.00 | 8.00 | 8.00 | 8.00 | 8.00 | 8.00 | 8.00 | 8.00 |
|  |  | A_h_ | 8.00 | 8.00 | 8.00 | 8.00 | 8.00 | 8.00 | 8.00 | 8.00 | 8.00 | 8.00 | 8.00 |
|  |  | B_l_ | 8.00 | 8.00 | 8.00 | 8.00 | 8.00 | 8.00 | 8.00 | 8.00 | 8.00 | 8.00 | 8.00 |
|  |  | B_m_ | 8.00 | 8.00 | 8.00 | 8.00 | 8.00 | 8.00 | 8.00 | 8.00 | 8.00 | 8.00 | 8.00 |
|  |  | B_h_ | 8.00 | 8.00 | 8.00 | 8.00 | 8.00 | 8.00 | 8.00 | 8.00 | 8.00 | 8.00 | 8.00 |
|  |  | C_l_ | 8.00 | 8.00 | 8.00 | 8.00 | 8.00 | 8.00 | 8.00 | 8.00 | 8.00 | 8.00 | 8.00 |
|  |  | C_m_ | 8.00 | 8.00 | 8.00 | 8.00 | 8.00 | 8.00 | 8.00 | 8.00 | 8.00 | 8.00 | 8.00 |
|  |  | C_h_ | 8.00 | 8.00 | 8.00 | 8.00 | 8.00 | 8.00 | 8.00 | 8.00 | 8.00 | 8.00 | 8.00 |
|  | Payment  Method  Balance | A_l_ | 4.30 | 4.22 | 4.00 | 3.62 | 3.10 | 2.42 | 1.60 | 0.62 | -0.50 | -1.78 | -3.20 |
|  |  | A_m_ | 7.16 | 7.09 | 6.86 | 6.49 | 5.96 | 5.29 | 4.46 | 3.49 | 2.36 | 1.09 | -0.34 |
|  |  | A_h_ | 10.03 | 9.95 | 9.73 | 9.35 | 8.83 | 8.15 | 7.33 | 6.35 | 5.23 | 3.95 | 2.53 |
|  |  | B_l_ | 4.50 | 4.43 | 4.20 | 3.83 | 3.30 | 2.63 | 1.80 | 0.83 | -0.30 | -1.58 | -3.00 |
|  |  | B_m_ | 7.50 | 7.43 | 7.20 | 6.83 | 6.30 | 5.63 | 4.80 | 3.83 | 2.70 | 1.43 | 0.00 |
|  |  | B_h_ | 10.50 | 10.43 | 10.20 | 9.83 | 9.30 | 8.63 | 7.80 | 6.83 | 5.70 | 4.43 | 3.00 |
|  |  | C_l_ | 4.73 | 4.65 | 4.43 | 4.05 | 3.53 | 2.85 | 2.03 | 1.05 | -0.08 | -1.35 | -2.78 |
|  |  | C_m_ | 7.88 | 7.80 | 7.58 | 7.20 | 6.68 | 6.00 | 5.18 | 4.20 | 3.08 | 1.80 | 0.38 |
|  |  | C_h_ | 11.03 | 10.95 | 10.73 | 10.35 | 9.83 | 9.15 | 8.33 | 7.35 | 6.23 | 4.95 | 3.53 |

Table S3 Experimental Parameters of Profit in Group IV (Under FFS)

| **Quantity (q)** | | **0** | **1** | **2** | **3** | **4** | **5** | **6** | **7** | **8** | **9** | **10** |
| --- | --- | --- | --- | --- | --- | --- | --- | --- | --- | --- | --- | --- |
| FFS-  Lev  -25% | A_l_ | 8.00 | 8.45 | 8.86 | 9.21 | 9.51 | 9.76 | 9.97 | 10.12 | 10.22 | 10.27 | 10.28 |
|  | A_m_ | 8.00 | 8.45 | 8.86 | 9.21 | 9.51 | 9.76 | 9.97 | 10.12 | 10.22 | 10.27 | 10.28 |
|  | A_h_ | 8.00 | 8.45 | 8.86 | 9.21 | 9.51 | 9.76 | 9.97 | 10.12 | 10.22 | 10.27 | 10.28 |
|  | B_l_ | 8.00 | 8.48 | 8.90 | 9.28 | 9.60 | 9.88 | 10.10 | 10.28 | 10.40 | 10.48 | 10.50 |
|  | B_m_ | 8.00 | 8.48 | 8.90 | 9.28 | 9.60 | 9.88 | 10.10 | 10.28 | 10.40 | 10.48 | 10.50 |
|  | B_h_ | 8.00 | 8.48 | 8.90 | 9.28 | 9.60 | 9.88 | 10.10 | 10.28 | 10.40 | 10.48 | 10.50 |
|  | C_l_ | 8.00 | 8.50 | 8.95 | 9.35 | 9.70 | 10.00 | 10.25 | 10.45 | 10.60 | 10.70 | 10.75 |
|  | C_m_ | 8.00 | 8.50 | 8.95 | 9.35 | 9.70 | 10.00 | 10.25 | 10.45 | 10.60 | 10.70 | 10.75 |
|  | C_h_ | 8.00 | 8.50 | 8.95 | 9.35 | 9.70 | 10.00 | 10.25 | 10.45 | 10.60 | 10.70 | 10.75 |
| FFS-  Lev  -50% | A_l_ | 8.00 | 8.91 | 9.71 | 10.42 | 11.02 | 11.53 | 11.93 | 12.24 | 12.44 | 12.54 | 12.55 |
|  | A_m_ | 8.00 | 8.91 | 9.71 | 10.42 | 11.02 | 11.53 | 11.93 | 12.24 | 12.44 | 12.54 | 12.55 |
|  | A_h_ | 8.00 | 8.91 | 9.71 | 10.42 | 11.02 | 11.53 | 11.93 | 12.24 | 12.44 | 12.54 | 12.55 |
|  | B_l_ | 8.00 | 8.95 | 9.80 | 10.55 | 11.20 | 11.75 | 12.20 | 12.55 | 12.80 | 12.95 | 13.00 |
|  | B_m_ | 8.00 | 8.95 | 9.80 | 10.55 | 11.20 | 11.75 | 12.20 | 12.55 | 12.80 | 12.95 | 13.00 |
|  | B_h_ | 8.00 | 8.95 | 9.80 | 10.55 | 11.20 | 11.75 | 12.20 | 12.55 | 12.80 | 12.95 | 13.00 |
|  | C_l_ | 8.00 | 9.00 | 9.90 | 10.70 | 11.40 | 12.00 | 12.50 | 12.90 | 13.20 | 13.40 | 13.50 |
|  | C_m_ | 8.00 | 9.00 | 9.90 | 10.70 | 11.40 | 12.00 | 12.50 | 12.90 | 13.20 | 13.40 | 13.50 |
|  | C_h_ | 8.00 | 9.00 | 9.90 | 10.70 | 11.40 | 12.00 | 12.50 | 12.90 | 13.20 | 13.40 | 13.50 |
| FFS-  Lev  -75% | A_l_ | 8.00 | 9.36 | 10.57 | 11.62 | 12.53 | 13.29 | 13.90 | 14.35 | 14.66 | 14.82 | 14.83 |
|  | A_m_ | 8.00 | 9.36 | 10.57 | 11.62 | 12.53 | 13.29 | 13.90 | 14.35 | 14.66 | 14.82 | 14.83 |
|  | A_h_ | 8.00 | 9.36 | 10.57 | 11.62 | 12.53 | 13.29 | 13.90 | 14.35 | 14.66 | 14.82 | 14.83 |
|  | B_l_ | 8.00 | 9.43 | 10.70 | 11.83 | 12.80 | 13.63 | 14.30 | 14.83 | 15.20 | 15.43 | 15.50 |
|  | B_m_ | 8.00 | 9.43 | 10.70 | 11.83 | 12.80 | 13.63 | 14.30 | 14.83 | 15.20 | 15.43 | 15.50 |
|  | B_h_ | 8.00 | 9.43 | 10.70 | 11.83 | 12.80 | 13.63 | 14.30 | 14.83 | 15.20 | 15.43 | 15.50 |
|  | C_l_ | 8.00 | 9.50 | 10.85 | 12.05 | 13.10 | 14.00 | 14.75 | 15.35 | 15.80 | 16.10 | 16.25 |
|  | C_m_ | 8.00 | 9.50 | 10.85 | 12.05 | 13.10 | 14.00 | 14.75 | 15.35 | 15.80 | 16.10 | 16.25 |
|  | C_h_ | 8.00 | 9.50 | 10.85 | 12.05 | 13.10 | 14.00 | 14.75 | 15.35 | 15.80 | 16.10 | 16.25 |

Table S4 Experimental Parameters of Profit in Group V (Under FFS)

| **Quantity (q)** | | | **0** | **1** | **2** | **3** | **4** | **5** | **6** | **7** | **8** | **9** | **10** |
| --- | --- | --- | --- | --- | --- | --- | --- | --- | --- | --- | --- | --- | --- |
| FFS-  Com  -25% | Fixed  wage | A_l_ | 8.00 | 8.91 | 9.71 | 10.42 | 11.02 | 11.53 | 11.93 | 12.24 | 12.44 | 12.55 | 12.55 |
|  |  | A_m_ | 8.00 | 8.91 | 9.71 | 10.42 | 11.02 | 11.53 | 11.93 | 12.24 | 12.44 | 12.55 | 12.55 |
|  |  | A_h_ | 8.00 | 8.91 | 9.71 | 10.42 | 11.02 | 11.53 | 11.93 | 12.24 | 12.44 | 12.55 | 12.55 |
|  |  | B_l_ | 8.00 | 8.95 | 9.80 | 10.55 | 11.20 | 11.75 | 12.20 | 12.55 | 12.80 | 12.95 | 13.00 |
|  |  | B_m_ | 8.00 | 8.95 | 9.80 | 10.55 | 11.20 | 11.75 | 12.20 | 12.55 | 12.80 | 12.95 | 13.00 |
|  |  | B_h_ | 8.00 | 8.95 | 9.80 | 10.55 | 11.20 | 11.75 | 12.20 | 12.55 | 12.80 | 12.95 | 13.00 |
|  |  | C_l_ | 8.00 | 9.00 | 9.90 | 10.70 | 11.40 | 12.00 | 12.50 | 12.90 | 13.20 | 13.40 | 13.50 |
|  |  | C_m_ | 8.00 | 9.00 | 9.90 | 10.70 | 11.40 | 12.00 | 12.50 | 12.90 | 13.20 | 13.40 | 13.50 |
|  |  | C_h_ | 8.00 | 9.00 | 9.90 | 10.70 | 11.40 | 12.00 | 12.50 | 12.90 | 13.20 | 13.40 | 13.50 |
|  | Payment  Method  Balance | A_l_ | 0.00 | 0.45 | 0.86 | 1.21 | 1.51 | 1.76 | 1.97 | 2.12 | 2.22 | 2.27 | 2.28 |
|  |  | A_m_ | 0.00 | 0.45 | 0.86 | 1.21 | 1.51 | 1.76 | 1.97 | 2.12 | 2.22 | 2.27 | 2.28 |
|  |  | A_h_ | 0.00 | 0.45 | 0.86 | 1.21 | 1.51 | 1.76 | 1.97 | 2.12 | 2.22 | 2.27 | 2.28 |
|  |  | B_l_ | 0.00 | 0.48 | 0.90 | 1.28 | 1.60 | 1.88 | 2.10 | 2.28 | 2.40 | 2.48 | 2.50 |
|  |  | B_m_ | 0.00 | 0.48 | 0.90 | 1.28 | 1.60 | 1.88 | 2.10 | 2.28 | 2.40 | 2.48 | 2.50 |
|  |  | B_h_ | 0.00 | 0.48 | 0.90 | 1.28 | 1.60 | 1.88 | 2.10 | 2.28 | 2.40 | 2.48 | 2.50 |
|  |  | C_l_ | 0.00 | 0.50 | 0.95 | 1.35 | 1.70 | 2.00 | 2.25 | 2.45 | 2.60 | 2.70 | 2.75 |
|  |  | C_m_ | 0.00 | 0.50 | 0.95 | 1.35 | 1.70 | 2.00 | 2.25 | 2.45 | 2.60 | 2.70 | 2.75 |
|  |  | C_h_ | 0.00 | 0.50 | 0.95 | 1.35 | 1.70 | 2.00 | 2.25 | 2.45 | 2.60 | 2.70 | 2.75 |
| FFS-  Com  -50% | Fixed  wage | A_l_ | 8.00 | 8.45 | 8.86 | 9.21 | 9.51 | 9.76 | 9.97 | 10.12 | 10.22 | 10.28 | 10.28 |
|  |  | A_m_ | 8.00 | 8.45 | 8.86 | 9.21 | 9.51 | 9.76 | 9.97 | 10.12 | 10.22 | 10.28 | 10.28 |
|  |  | A_h_ | 8.00 | 8.45 | 8.86 | 9.21 | 9.51 | 9.76 | 9.97 | 10.12 | 10.22 | 10.28 | 10.28 |
|  |  | B_l_ | 8.00 | 8.48 | 8.90 | 9.28 | 9.60 | 9.88 | 10.10 | 10.28 | 10.40 | 10.48 | 10.50 |
|  |  | B_m_ | 8.00 | 8.48 | 8.90 | 9.28 | 9.60 | 9.88 | 10.10 | 10.28 | 10.40 | 10.48 | 10.50 |
|  |  | B_h_ | 8.00 | 8.48 | 8.90 | 9.28 | 9.60 | 9.88 | 10.10 | 10.28 | 10.40 | 10.48 | 10.50 |
|  |  | C_l_ | 8.00 | 8.50 | 8.95 | 9.35 | 9.70 | 10.00 | 10.25 | 10.45 | 10.60 | 10.70 | 10.75 |
|  |  | C_m_ | 8.00 | 8.50 | 8.95 | 9.35 | 9.70 | 10.00 | 10.25 | 10.45 | 10.60 | 10.70 | 10.75 |
|  |  | C_h_ | 8.00 | 8.50 | 8.95 | 9.35 | 9.70 | 10.00 | 10.25 | 10.45 | 10.60 | 10.70 | 10.75 |
|  | Payment  Method  Balance | A_l_ | 0.00 | 0.91 | 1.71 | 2.42 | 3.02 | 3.53 | 3.93 | 4.24 | 4.44 | 4.54 | 4.55 |
|  |  | A_m_ | 0.00 | 0.91 | 1.71 | 2.42 | 3.02 | 3.53 | 3.93 | 4.24 | 4.44 | 4.54 | 4.55 |
|  |  | A_h_ | 0.00 | 0.91 | 1.71 | 2.42 | 3.02 | 3.53 | 3.93 | 4.24 | 4.44 | 4.54 | 4.55 |
|  |  | B_l_ | 0.00 | 0.95 | 1.80 | 2.55 | 3.20 | 3.75 | 4.20 | 4.55 | 4.80 | 4.95 | 5.00 |
|  |  | B_m_ | 0.00 | 0.95 | 1.80 | 2.55 | 3.20 | 3.75 | 4.20 | 4.55 | 4.80 | 4.95 | 5.00 |
|  |  | B_h_ | 0.00 | 0.95 | 1.80 | 2.55 | 3.20 | 3.75 | 4.20 | 4.55 | 4.80 | 4.95 | 5.00 |
|  |  | C_l_ | 0.00 | 1.00 | 1.90 | 2.70 | 3.40 | 4.00 | 4.50 | 4.90 | 5.20 | 5.40 | 5.50 |
|  |  | C_m_ | 0.00 | 1.00 | 1.90 | 2.70 | 3.40 | 4.00 | 4.50 | 4.90 | 5.20 | 5.40 | 5.50 |
|  |  | C_h_ | 0.00 | 1.00 | 1.90 | 2.70 | 3.40 | 4.00 | 4.50 | 4.90 | 5.20 | 5.40 | 5.50 |
| FFS-  Com  -75% | Fixed  wage | A_l_ | 8.00 | 8.00 | 8.00 | 8.00 | 8.00 | 8.00 | 8.00 | 8.00 | 8.00 | 8.00 | 8.00 |
|  |  | A_m_ | 8.00 | 8.00 | 8.00 | 8.00 | 8.00 | 8.00 | 8.00 | 8.00 | 8.00 | 8.00 | 8.00 |
|  |  | A_h_ | 8.00 | 8.00 | 8.00 | 8.00 | 8.00 | 8.00 | 8.00 | 8.00 | 8.00 | 8.00 | 8.00 |
|  |  | B_l_ | 8.00 | 8.00 | 8.00 | 8.00 | 8.00 | 8.00 | 8.00 | 8.00 | 8.00 | 8.00 | 8.00 |
|  |  | B_m_ | 8.00 | 8.00 | 8.00 | 8.00 | 8.00 | 8.00 | 8.00 | 8.00 | 8.00 | 8.00 | 8.00 |
|  |  | B_h_ | 8.00 | 8.00 | 8.00 | 8.00 | 8.00 | 8.00 | 8.00 | 8.00 | 8.00 | 8.00 | 8.00 |
|  |  | C_l_ | 8.00 | 8.00 | 8.00 | 8.00 | 8.00 | 8.00 | 8.00 | 8.00 | 8.00 | 8.00 | 8.00 |
|  |  | C_m_ | 8.00 | 8.00 | 8.00 | 8.00 | 8.00 | 8.00 | 8.00 | 8.00 | 8.00 | 8.00 | 8.00 |
|  |  | C_h_ | 8.00 | 8.00 | 8.00 | 8.00 | 8.00 | 8.00 | 8.00 | 8.00 | 8.00 | 8.00 | 8.00 |
|  | Payment  Method  Balance | A_l_ | 0.00 | 1.36 | 2.57 | 3.62 | 4.53 | 5.29 | 5.90 | 6.35 | 6.66 | 6.82 | 6.83 |
|  |  | A_m_ | 0.00 | 1.36 | 2.57 | 3.62 | 4.53 | 5.29 | 5.90 | 6.35 | 6.66 | 6.82 | 6.83 |
|  |  | A_h_ | 0.00 | 1.36 | 2.57 | 3.62 | 4.53 | 5.29 | 5.90 | 6.35 | 6.66 | 6.82 | 6.83 |
|  |  | B_l_ | 0.00 | 1.43 | 2.70 | 3.83 | 4.80 | 5.63 | 6.30 | 6.83 | 7.20 | 7.43 | 7.50 |
|  |  | B_m_ | 0.00 | 1.43 | 2.70 | 3.83 | 4.80 | 5.63 | 6.30 | 6.83 | 7.20 | 7.43 | 7.50 |
|  |  | B_h_ | 0.00 | 1.43 | 2.70 | 3.83 | 4.80 | 5.63 | 6.30 | 6.83 | 7.20 | 7.43 | 7.50 |
|  |  | C_l_ | 0.00 | 1.50 | 2.85 | 4.05 | 5.10 | 6.00 | 6.75 | 7.35 | 7.80 | 8.10 | 8.25 |
|  |  | C_m_ | 0.00 | 1.50 | 2.85 | 4.05 | 5.10 | 6.00 | 6.75 | 7.35 | 7.80 | 8.10 | 8.25 |
|  |  | C_h_ | 0.00 | 1.50 | 2.85 | 4.05 | 5.10 | 6.00 | 6.75 | 7.35 | 7.80 | 8.10 | 8.25 |

Table S5 Experimental Parameters of Patient Benefit

| **Quantity (q)** | | **0** | **1** | **2** | **3** | **4** | **5** | **6** | **7** | **8** | **9** | **10** |
| --- | --- | --- | --- | --- | --- | --- | --- | --- | --- | --- | --- | --- |
| Patient  Benefit | A_l_ | 7 | 8 | 9 | 10 | 9 | 8 | 7 | 6 | 5 | 4 | 3 |
|  | A_m_ | 5 | 6 | 7 | 8 | 9 | 10 | 9 | 8 | 7 | 6 | 5 |
|  | A_h_ | 3 | 4 | 5 | 6 | 7 | 8 | 9 | 10 | 9 | 8 | 7 |
|  | B_l_ | 12 | 13 | 14 | 15 | 14 | 13 | 12 | 11 | 10 | 9 | 8 |
|  | B_m_ | 10 | 11 | 12 | 13 | 14 | 15 | 14 | 13 | 12 | 11 | 10 |
|  | B_h_ | 8 | 9 | 10 | 11 | 12 | 13 | 14 | 15 | 14 | 13 | 12 |
|  | C_l_ | 14 | 16 | 18 | 20 | 18 | 16 | 14 | 12 | 10 | 8 | 6 |
|  | C_m_ | 10 | 12 | 14 | 16 | 18 | 20 | 18 | 16 | 14 | 12 | 10 |
|  | C_h_ | 6 | 8 | 10 | 12 | 14 | 16 | 18 | 20 | 18 | 16 | 14 |


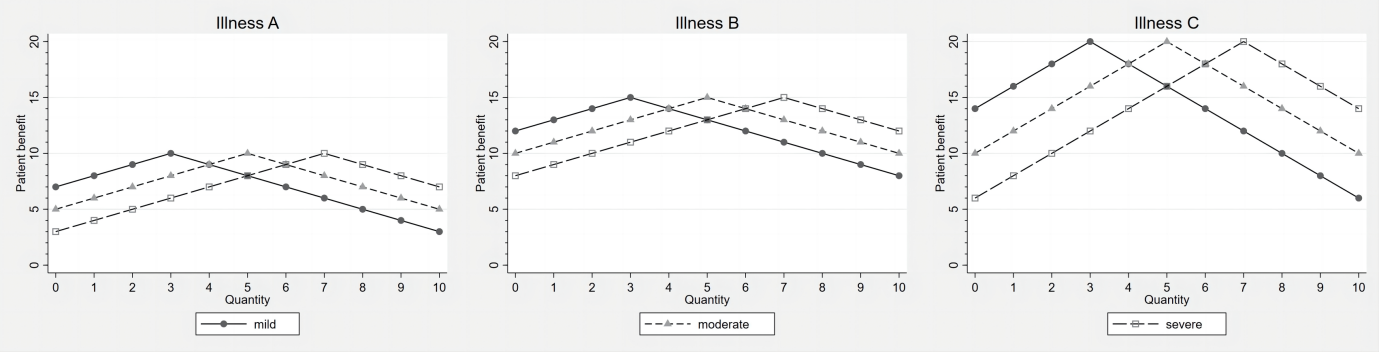


Fig. 1 Patient Benefit of Different Illness Type and Severity of Illness

Notes: Fig. 1 depicts patient benefit parameters for illnesses A, B, and C across different severity on the quantity interval from 0 to 10. From left to right, the panels indicate patient benefit for illnesses A, B, and C, respectively. The marginal patient benefit for illnesses A and B is 1, but for illness C is 2. The solid line represents severity mild, the dashed line represents severity moderate, and the long-dashed line represents severity severe. The patient benefit remains constant in all experimental conditions.
