# Supplementary material for: The effect of internal salary incentives based on insurance payment on physicians’ behavior: experimental evidence
Source: BMC Health Serv Res. 2023 Dec 14;23:1410. doi: 10.1186/s12913-023-10408-8 (PMC10720113; doi:10.1186/s12913-023-10408-8)
Supplement: Supplementary file 2 — Additional file 2. Instructions + Comprehension Questions. [file 12913_2023_10408_MOESM2_ESM.docx]

**Additional File 2: Instructions + Comprehension Questions**

Thank you for participating in the experiment on time! You are participating in an economic experiment on decision-making behavior. In the experiment, in a role of physician, you will provide medical services for different types of patients under different salaries. The experiment will last for about 90 minutes and consists of three parts. Your decision in any part of the experiment will not affect the other parts of the experiment. According to your decisions, you will receive a payment after the experiment.

Please read the following instructions carefully. If you have any questions, please raise your hand and we will answer any questions you may have. Please do not talk to others during the experiment. Thank you for your cooperation!

1 Experimental Situations

In the experiment, you will take on the role of a physician and choose the quantity of medical services for different types of patients. The types of patients are characterized by one of three illnesses (A, B, C), and each illness may occur in three different degrees of severity (l, m, h, i.e. mild, moderate and severe). In each decision-making task, you will face one patient, who is characterized by one of nine combinations of different illnesses and severities (A_l_, B_l_, C_l_, A_m_, B_m_, C_m_, A_h_, B_h_, C_h_). Your decision is to provide the patient with a quantity of 0, 1, 2, 3, 4, 5, 6, 7, 8, 9 or 10 medical services.

You will receive a fixed wage for your decisions, but the fixed wage in each part of the experiment is different. You will incur the cost of treating patients, which depends on the quantity of medical services you provide. However, you don’t need to bear the costs.

Every quantity of medical service yields a specific patient benefit, depending on the patients’ illness and severity. Therefore, your decisions not only affect your profit, but also the patient benefit.

2 Payment

All amounts in the experiment are presented in Talers. 1 Taler equals 0.04 CNY.

After the experiment, the basic remuneration, 30 CNY, and your profit from all the experimental decisions will be paid to you by bank transfer.

Although there are no real patients in the experiment, your decisions do benefit real patients. One of the 5 rounds of the experiment will be chosen randomly. The sum of patient benefits in this round will be donated to the Red Cross Society of China. The donation will be carried out by the experimenter and one monitor that we select from the subjects randomly. Also, this monitor will receive an additional 50 CNY for remuneration.

3 Experimental Steps

Step 1: Experiment Introductions. Please read the experimental instructions. If you have any questions, please raise your hand. Before the formal experiment, we will have a pilot experiment to make you get familiar with this experiment.

Step 2: Experiment Decisions. According to the information presented on the computer screen, you need to choose the quantity of medical services for different types of patients within the specified time. Your operation is to enter a non-negative integer whose value is 0, 1, 2, 3, 4, 5, 6, 7, 8, 9 or 10 on the computer screen.

Step 3: Experiment Payments. Your profit and patient benefit of each decision will be displayed on the computer screen.

Step 4: Experiment Rounds. The experiment consists of five rounds and each round includes 27 decisions. When all participants complete the current experimental decision, the experiment automatically enters to the next decision. Repeat the above operations until all experimental decisions are completed.

Pilot Experiment Instructions:

The pilot experiment consists of two parts. In Part I, you will need to answer some comprehension questions.

Experimental Interface 1 in Part I: in the upper left of the experiment interface, it shows the patient type corresponding to each decision, such as “Patient Type: A_l_”. In the middle part of the experiment interface, it presents various information, “Your Cost”, “Your Salary” and “Patient Benefit”, which are corresponding to different quantity of medical service you may provide.

The questions you need to answer are as follows.

Assuming that the quantity of medical service you provide is 4, then:

(1) What is the “Your Cost”?

(2) What is the “Your Salary”?

(3) What is the “Patient Benefit”?

Please fill in the answers to the above questions in the three boxes at the bottom of the experiment interface according to the information on the computer screen. If your answers are correct, after clicking the “OK” button at the bottom right corner, the experiment interface will automatically enter to the Experimental Interface 2 in Part I. Otherwise, please re-answer until your answer is correct.

Experimental Interface 2 in Part I: the information on the computer screen is the same as that in Experimental Interface 1 in Part I.

The questions you need to answer are as follows.

(1) How much salary can you get according to the quantity of medical service you provide?

(2) When the “Patient Benefit” is the lowest, what is the quantity of medical service you need to provide?

(3) When the “Patient Benefit” is the highest, what is the quantity of medical service you need to provide?

Please fill in the answers to the above questions in the three boxes at the bottom of the experiment interface according to the information on the computer screen. If your answers are correct, after clicking the “OK” button at the bottom right corner, the experiment interface will automatically enter to the Part II. Otherwise, please re-answer until your answer is correct.

Part II of pilot experiment: the simulation of formal experiment.

In the formal experiment, you firstly need to fill in your corresponding experiment number. The prompt content on the computer screen is “What is your experiment number” (Everyone corresponds to a specific experiment number. Be careful not to fill in the wrong number).

After filling in the experiment number, you will enter to the experiment interface. In the upper left part of the experimental interface, it will introduce the patient type of each decision, such as “Patient Type: A_l_”. In the middle part of the experiment interface, it presents various information, “Your Cost”, “Your Salary”, and “Patient Benefit”, which corresponds to the quantity of medical service you may provide. According to such information, you need to choose the quantity of medical service. A prompt will appear at the bottom of the experiment interface, “Please indicate the quantity of medical service you wish to provide”. Please enter the quantity of medical service (0, 1, 2, 3, 4, 5, 6, 7, 8, 9 or 10) you want to provide in the box behind the prompt.

After entering the quantity number, when you click the “OK” button at the bottom right corner of the computer screen, the experiment interface will automatically enter to the experiment result output interface. At this time, two lines of content will appear on the computer screen. The first line is “Your Salary is XX”, and the second line is “Patient Benefit is XX”. In the formal experiment, you need to fill in these data into the corresponding place in the experiment record form. After that, click the “OK” button at the bottom right corner of the computer screen and you will automatically enter to the next decision.
